# Core-binding factor beta is required for osteoblast differentiation during fibula fracture healing

**DOI:** 10.1186/s13018-021-02410-9

**Published:** 2021-05-14

**Authors:** Tuanmao Guo, Yanli Xing, Zhongning Chen, Xianhong Wang, Haiyun Zhu, Lan Yang, Yong Yan

**Affiliations:** 1 The Second Department of Orthopedics, Xianyang Central Hospital, Xianyang, 712000 People’s Republic of China; 2 The Pharmacy Department, Xianyang Central Hospital, Xianyang, 712000 People’s Republic of China; 3 The Second Department of Orthopedics, Shaanxi Traditional Chinese Medicine Hospital, Xi’an, 710003 People’s Republic of China

**Keywords:** Core-binding factor beta, Fracture healing, Osteoblast, Differentiation, Knockout

## Abstract

**Background:**

Growing evidence has implicated core-binding factor beta (Cbfb) as a contributor to osteoblast differentiation, which plays a key role in fracture healing. Herein, we aimed to assess whether Cbfb affects osteoblast differentiation after fibula fracture.

**Methods:**

Initially, we established a Cbfb conditional knockout mouse model for subsequent studies. Immunohistochemical staining was conducted to detect the expression of proliferating cell nuclear antigen (PCNA) and collagen II in the fracture end. Next, we isolated and cultured osteoblasts from specific Cbfb conditional knockout mice for BrdU analysis, alkaline phosphatase (ALP) staining, and von Kossa staining to detect osteoblast viability, differentiation, and mineralization, respectively. Western blot analysis and reverse transcription-quantitative polymerase chain reaction (RT-qPCR) were used to detect the expression of osteoblast differentiation-related genes.

**Results:**

The Cbfb conditional knockout mice exhibited downregulated expression of PCNA and collagen II, reduced ALP activity, and mineralization, as well as diminished expression of osteoblast differentiation-related genes. Further, Cbfb knockout exerted no obvious effects on osteoblast proliferation.

**Conclusions:**

Overall, these results substantiated that Cbfb could promote fibula fracture healing and osteoblast differentiation and thus provided a promising therapeutic target for clinical treatment of fibula fracture.

**Supplementary Information:**

The online version contains supplementary material available at 10.1186/s13018-021-02410-9.

## Background

Fibula fracture is a relatively common long bone fracture, which often leads to soft tissue complications and non-union [1]. As such, fibula fracture represents a recurring injury that often needs stabilization through orthopedic surgery approaches [2]. Fracture healing is a complicated biological process since it entails specific regenerative patterns and involves altered expression of several thousand genes [3]. The procedure of preemptive anatomical reduction and fixation can facilitate the healing of fibula fracture and restoration of lower-extremity alignment [4]. Fractures that cannot be made to heal or unite quickly and completely are responsible for pain and loss of mobility [5]. Osteoblasts, which originate from mesodermal progenitors, are essential for bone-forming and mineralization processes [6]. Osteoblasts express receptors for several hormones including estrogen, parathormone (PTH), and glucocorticoids, all of which participate in the regulation of osteoblast differentiation [7], and are necessary for normal fracture healing [8].

Core-binding factor beta (Cbfb), also known as polyomavirus enhancer binding protein 2 beta gene and SL3 enhancer factor 1, can fuse to form a chimeric protein [9]. Cbfb is essential for embryonic bone morphogenesis and postnatal cartilage and bone formation, through its control of the balance of chondrocyte differentiation and proliferation via regulation of Indian hedgehog expression and parathyroid hormone-related protein receptor [10]. Cbfb knockout mice present with severely delayed bone formation, which is characterized by diminished osteoblast differentiation and maturation and impaired skeletal development [10]. Similar findings revealed by Fukuda et al. showed that Cbfb favored osteoblast differentiation and bone formation [11]. However, the role of Cbfb in fracture healing remains to be substantiated, whereas other targets, such as C1q/TNF-related protein 3 (CTRP3) and nuclear factor erythroid 2-related factor 2 (Nrf2), have been highlighted in fracture healing based on their involvement in endochondral ossification and osteoblast metabolism [12–14], indicating the great potential of Cbfb for fracture healing management. In addition, Cbfb has been reported to modulate bone development through stabilizing runt-related transcription factor (Runx) family proteins, and Runx2 serves as a critical target in fracture healing [15–17]. Given the aforementioned evidence, we conducted this study to ascertain specific mechanistic actions of Cbfb in fibula fracture healing via regulation of osteoblast differentiation in an animal model.

## Materials and methods

### Ethics statement

All experimental procedures were in strict accordance with the requirements of the Animal Ethics Committee of Xianyang Central Hospital. Extensive efforts were made to ensure minimal suffering of the animals used during the study.

### Construction of Cbfb conditional knockout mouse model

Loxp was inserted at both ends of the Cbfb allele to construct Cbfb fluorine and oxygen (Flox) mice (Jackson Lab, CA, USA), which were hybridized with Dermo promoter Cre (Cyclization Recombination Enzyme) mice (Jackson Lab, CA, USA), and the first filial generation Cbfbf/f; Dermo-Cre mice were obtained. These first filial generation mice were hybridized with Cbfb Flox mice, yielding the second generation of experimental group (Cbfbf/f; Dermo-Cre mice) with specific knockdown of Cbfb in mesenchymal stem cells. The littermate Cbfbf/f and Cbfbf/f*+* mice were taken as the controls of wild type (WT) mice.

### Identification of Cbfb conditional knockout in mouse genotype

Mice at postnatal age of 2–3 weeks were selected and the tips of their tails (2–3 mm) were collected and placed in 0.2 mL mouse tail digestive juice added with 4 μL proteinase K solution (Yeasen Biotechnology Co., Ltd., Shanghai, China) and incubated overnight at 55°C with shaking. Next, the samples were added with 0.1 mL 6 M NaOH solution (Guangzhou Laiyu Chemical Co., Ltd, Guangzhou, China), shaken violently and centrifuged. The supernatant was added to 0.5 mL of 95% ethanol (Sinopharm Chemical Reagent Co., Ltd., Beijing China), shaken up and down 35 times, and allowed to stand at room temperature for 10 min, followed by centrifugation for 5 min. Then, the precipitate was washed with 1 mL of 75% ethanol for 15 s and dried, whereupon 0.1 mL 0.1 TE solution (pH = 8.0; Shanghai Haling Biotechnology Co., Ltd., Shanghai, China) was added and shaken for 1 h at 220 rpm and 65°C to dissolve the DNA. The forward sequence of Cbfb identification primers was 5′-TGTCTGAAGACAACTACAGTGTAC-3′, and the reverse sequence was 5′-CTCTCTGAACACTATATCAGTTCC-3′. The forward sequence of CRE (Cyclization Recombination Enzyme) identification primers was 5′-CCTGGAAAATGCTTCTGTCCGTTTGCC-3′, and the reverse sequence was 5′-GAGTTGATAGCTGGCTGGTGGCAGATG-3′. The reaction conditions of reverse transcription quantitative polymerase chain reaction (RT-qPCR) consisted of pre-denaturation at 94°C for 5 min, denaturation at 94°C for 45 s, annealing at 54°C (CRE) and 57°C (Cbfb) for 45 s, extension at 72°C for 45 s, with a total of 30 cycles, and elongation at 72°C for 5 min. The PCR was performed in a 2720 thermal cycler (Applied Biosystems, Thermo Fisher Scientific Inc., Waltham, MA, USA). The PCR products were separated using 1.0% agarose gel electrophoresis and photographed under an ultraviolet imaging system. The position of PCR product bands served to judge the size of the product fragments and then determine the genotypes of the mice.

### Establishment of fracture healing model

A total of 30 7-week-old mice were divided into control and experimental groups (*n* = 15). The mice were fixed on a homemade autopsy table. An incision about 5-mm long was made along the proximal part of right lateral leg 12-mm away from the bone nodule. The soft tissues and muscles surrounding the fibula were separated by blunt dissection, and bleeding was staunched for exposure of the fibula, followed by transverse shearing. Mice with a deformity of the fracture or otherwise failing to meet the model requirements were excluded. Five mice for each group were subsequently euthanized at days 1, 14, and 28 after injury (Supplementary figure 1). Here, the fracture end and its surrounding tissues were collected for subsequent experiments.

### Streptavidin-perosidase (SP) assay

The specimens rinsed using normal saline and then fixed in 4% paraformaldehyde (pH = 7.4, Beijing Cellchip Biotechnology Co., Ltd., Beijing, China) at 4°C for 24–48 h. Next, decalcification was conducted by incubation in 200 g/L ethylenediamine tetraacetic acid (EDTA) (Baoding Kaiyue Chemical Co., Ltd., Hebei, China) for 4 weeks, with replacement of the decalcification fluid every 3 days. Next, the specimens were dehydrated with gradient ethanol, cleared with xylene (Changsha Tang Hua Chemical Trading Co., Ltd., Changsha, China) and vertically embedded in paraffin. The paraffin-embedded specimens were placed in a 4°C refrigerator and later sectioned and stained using Safranin-O/Fast Green for microscopic examination of the fracture healing site. Immunohistochemical staining was carried out to detect the expression of proliferating cell nuclear antigen (PCNA) and collagen II in the fracture end. The stained slices were observed under an optical microscope (XSP-36, Boshida Optical Instruments, Shenzhen, China) in five randomly selected fields of view per slice, with counting of 100 cells in each field of view to calculate the average proportion of positive cells. The experiment was conducted in triplicate. The primary antibody PCNA and collagen II was purchased from Abcam Inc. (Cambridge, MA, USA) and the secondary antibody was purchased from Shanghai MICROTEK Biotechnology Co., Ltd. (Shanghai, China).

### Cell culture and detection of cell proliferation and differentiation

Osteoblast precursor cells were isolated and then inoculated into a cell culture dish at a density of 3 × 10^3^ cells/cm^2^. Upon reaching confluence, the cells were added with osteoblast induction medium: BGJB medium (Thermo Fisher Scientific Inc., Waltham, MA, USA) containing 10% fetal bovine serum (FBS), 50 μg/mL l-ascorbic acid (A4544, Sigma-Aldrich Chemical Company, St Louis, MO, USA) and 5 μg β-glycerolphosphate (G9891, Sigma-Aldrich Chemical Company, St Louis, MO, USA). Then, the 5-bromo-2′-deoxyuridine (BrdU) labeling method was used to measure osteoblast proliferation. Next, the cells were colored using 3,3′-diaminobenzidine (DAB) and observed under a microscope, with positive cells presenting with brownish yellow color. At the 14th day, alkaline phosphatase (ALP) staining (A2356, Sigma-Aldrich Chemical Company, St Louis, MO, USA) was adopted to detect cell differentiation. At the 21st day, von Kossa staining (Thermo Fisher Scientific Inc., Waltham, MA, USA) was applied to test the mineralization of osteoblasts.

### RNA isolation and quantitation

Total RNA was extracted from primary osteoblasts using Trizol. Then, the reverse transcription was conducted using Invitrogen SuperScript VILO Master Mix kit (Thermo Fisher Scientific Inc., Waltham, MA, USA). Then, RT-qPCR was performed on a PCR instrument (Applied Bio-systems, Foster City, CA, USA) using SYBR Green (Thermo Fisher Scientific Inc., Waltham, MA, USA). The primers used are shown in Supplementary Table 1.

### Western blot analysis

The cells were rinsed twice with phosphate-buffered saline (PBS) and then added with loading buffer for heating at 95°C for 10 min. The proteins were separated by 10% polyacrylamide gel electrophoresis (Boster Biological Technology Co., Ltd., Wuhan, Hubei, China) (40 μg/well) and then transferred onto polyvinylidene fluoride (PVDF) membranes using the wet transfer method. The membranes were blocked with 5% bovine serum albumin (BSA) at room temperature for 1 h and then incubated with primary antibodies diluted at 1: 500–1: 1000: Osteocalcin, (AM0911, Millipore, Billerica, MA, USA), Osterix, (ab22552, Abcam Inc., Cambridge, MA, USA) and ATF4 (abl05383, Abcam Inc., Cambridge, MA, USA) at 4°C overnight. After being rinsed three times (5 min/time) with Tris-buffered saline Tween-20 (TBST), the membranes were added with the corresponding secondary antibody (Abcam Inc., Cambridge, MA, USA) (Shanghai Precision & Scientific Instrument Co., Ltd., Shanghai, China) and incubated at room temperature for 1 h, followed by three washes with TBST (5 min/time). The immunocomplexes on the membrane were visualized using enhanced chemiluminescence (ECL) reagent (Shanghai Genmed Gene Pharmaceutical Technology Co., Ltd., Shanghai, China) and band intensities were quantified using a multifunctional imaging system.

### Statistical analysis

Statistical analyses were conducted using SPSS 19.0 statistical software (IBM Corp. Armonk, NY, USA). Measurement data were expressed as mean ± standard deviation and compared by unpaired *t* test between two groups. A *p* < 0.05 demonstrated statistical significance.

## Results

### Identification of Cbfb conditional knockout mice

**Fig. 1:**
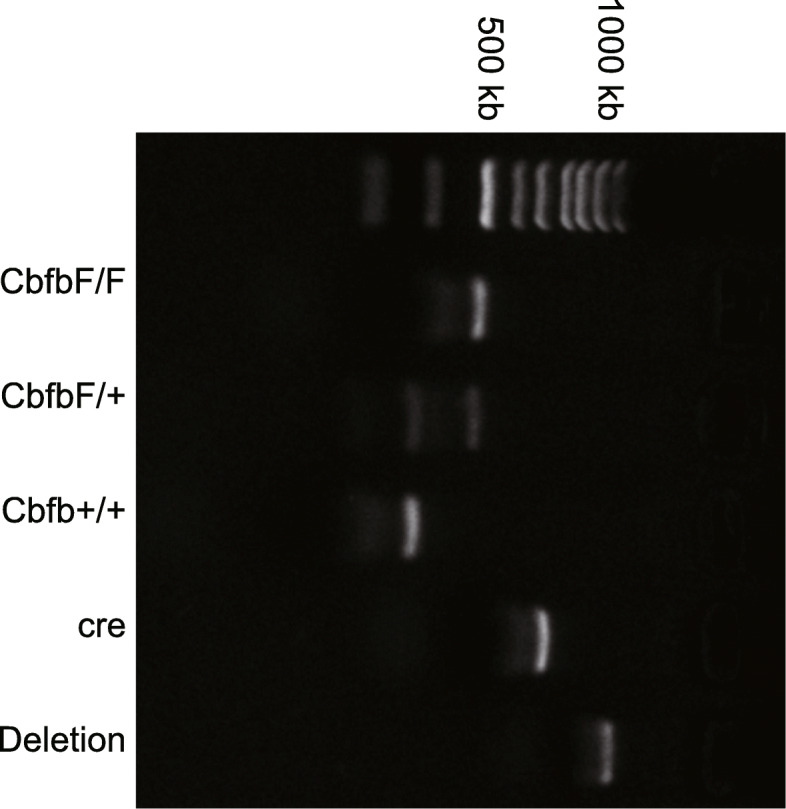
Identification of Cbfb conditional knockout mice

After extracting the DNA of tails of mice following Cbfb conditional knockout, we used RT-qPCR to identify mouse genotypes, including WT, knocked out of fifth exon of Cbfb, insertion of Loxp fragments at both ends of the Cbfb allele, and the sequence with CRE recombinase, as illustrated in Fig. 1.

### Histological observation of morphological characteristics of Cbfbf/f; Dermo-Cre mice

**Fig. 2:**
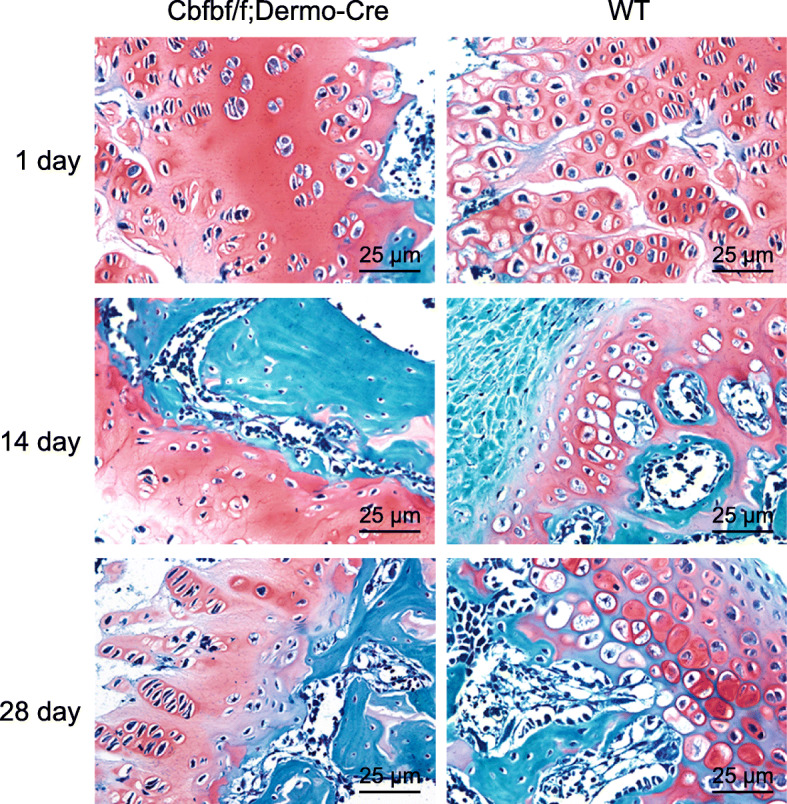
Observation of morphological characteristics of Cbfbf/f; Dermo-Cre mice (× 400). Left panels, morphology of healed tissues of Cbfbf/f; Dermo-Cre mice at days 1, 14, and 28 following fracture. Right panels, morphology of healed tissues of WT mice at days 1, 14, and 28 following fracture

At the 1st day after fracture, the staining results revealed that most fracture ends of Cbfbf/f; Dermo-Cre mice and WT mice were filled with fibrous tissues. At the 14th day after fracture, chondrocytes at the fracture end of WT mice were significantly decreased in number and hypertrophic and part of the cartilage callus had turned into bony callus. However, the number of chondrocytes of Cbfbf/f; Dermo-Cre mice had increased significantly and now formed a large proportion of the cartilage callus. At the 28th day after fracture, the fractures of WT mice were healed, accompanied by regularly distributed bone trabecula. However, the bone trabecula of Cbfbf/f; Dermo-Cre mice were observed to have a disordered arrangement, and the fracture healing was arrested at the shaping stage, with a paucity of chondrocytes (Fig. 2).

### Cbfb knockout decreases expression of PCNA and collagen II

**Fig. 3:**
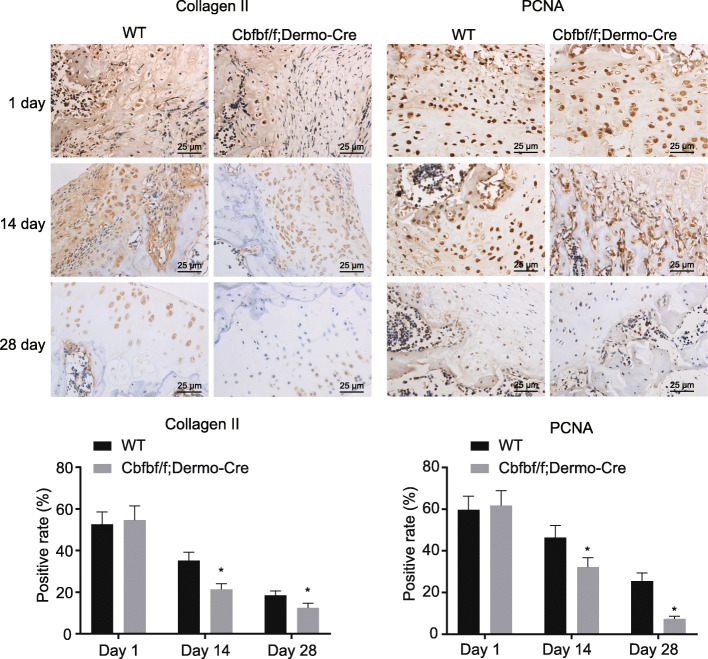
Cbfb knockout decreases expression of collagen II (× 400) and PCNA (× 400). Measurement data were expressed as mean ± standard deviation. Data comparison was conducted using independent samples *t* test. *p < 0.05 versus the WT group. *n* = 3

Immunohistochemical staining analysis of Collagen II (Fig. 3 left panel) revealed no obvious difference between the collagen II expression of the WT and the Cbfbf/f; Dermo-Cre mice on the 1st day after the fracture. On the 14th and 28th days, the fractured ends of the WT mice were mostly composed of chondrocytes, but only a few chondrocytes were observed in the Cbfbf/f; Dermo-Cre mice. At day 14 and 28, the collagen II expression was downregulated in the WT and Cbfbf/f; Dermo-Cre mice, whereas the latter presented more significant downregulation. Meanwhile, immunohistochemical staining analysis of PCNA (Fig. 3 right panel) showed that on first days after fracture, the fracture end was mainly composed of undifferentiated fibroblasts and cartilage precursor cells. The positive expression of PCNA was observed to be diminished both in the WT and Cbfbf/f; Dermo-Cre groups. On the 14th and 28th days after fracture, the number of cells in the proliferative phase of fracture ends was decreased, and the PCNA positive expression was down-regulated in the WT mice and the Cbfbf/f; Dermo-Cre mice, whereas the latter exhibited much more distinct downregulation.

### Cbfb knockout inhibits ALP activity and mineralization

**Fig. 4:**
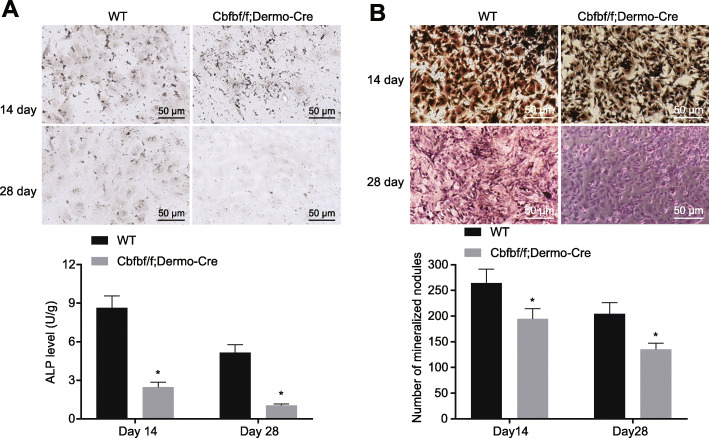
Cbfb knockout inhibits ALP activity and mineralization in osteoblasts. **a** ALP staining (× 200) analysis of ALP activity; **b** von Kossa staining (× 200) analysis of mineralization of osteoblasts. Measurement data were expressed as mean ± standard deviation. Data comparison was conducted using independent samples *t* test. * *p* < 0.05 versus the WT group. *n* = 3

The results obtained from ALP staining and von Kossa staining indicated that the activity of ALP in osteoblasts was decreased in the Cbfbf/f; Dermo-Cre group, and osteoblast differentiation was inhibited (Fig. 4a). The calcium nodules of osteoblasts in the Cbfbf/f; Dermo-Cre mice were much fewer/smaller than those in the WT mice, and the extent of mineralization was inhibited (Fig. 4b).

### Cbfb knockout has no effects on osteoblast proliferation

**Fig. 5:**
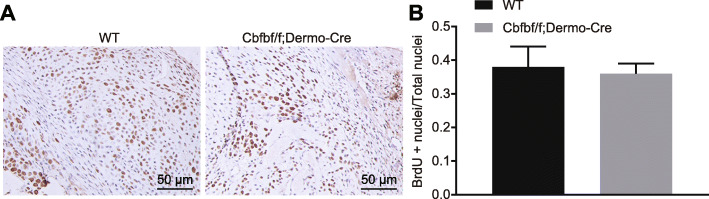
Cbfb knockout exhibits few effects on osteoblast proliferation. **a** BrdU assay (× 200) for osteoblast proliferation. **b** The proportion of BrdU-positive cells at panel **a**

Subsequent BrdU results showing yellow-brown proliferating cells, with no statistically significant difference in the proportion of positive cells between the Cbfbf/f; Dermo-Cre mice and the WT group (*p* > 0.05) (Fig. 5). This indicates that Cbfb knockout did not affect osteoblast proliferation.

### Cbfb knockout decreases osteoblast differentiation

**Fig. 6:**
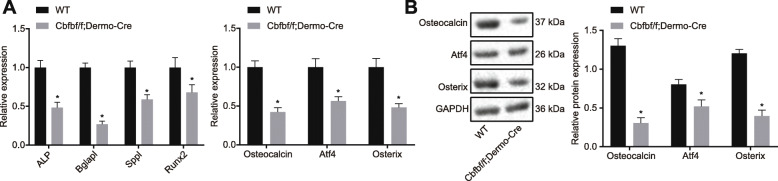
Cbfb knockout represses osteoblast differentiation. **a** mRNA expression of ALP, Bglapl, SPPL, Runx2, Osteocalcin, Atf4, and Osterix determined by RT-qPCR; **b** Western blot analysis of Osteocalcin, Atf4, and Osterix proteins

On the 14th day after the fracture, RT-qPCR assay revealed lower mRNA expression of the osteoblast differentiation-related genes, including ALP, Bglapl, Runx2, and SPPL, in the Cbfbf/f; Dermo-Cre mice than that in the WT mice (*p* < 0.05) (Fig. 6a). Western blot analysis showed varying degrees of reduced protein expression of the osteoblast differentiation-related genes Osteocalcin, Atf4, and Osterix in osteoblasts after Cbfb knockout, indicating inhibition of osteoblast differentiation. Compared with the WT mice, the mRNA and protein expression of osteocalcin, Atf4, and Osterix in the Cbfbf/f; Dermo-Cre mice were reduced to different degrees (Fig. 6b).

## Discussion

Fracture healing represents a unique biologic process that begins with an initial inflammatory response, during which bone and the immune system interact with each other closely [18]. CBFβ serves as a non-DNA-binding partner of all Runx proteins and is essential for transcription activity of CBF transcription factors [19]. A growing number of studies have recognized the Cbfb gene to be a critical factor in bone morphogenesis, but the underlying molecular mechanisms remain largely unknown [10, 20, 21]. In this study, we confirmed that Cbfb expression was associated with fibula fracture healing and osteoblast differentiation. The main findings of our study indicated that Cbfb could potentially promote fibula fracture healing and osteoblast differentiation.

The shortened limbs and lower weight of Cbfbf/f; Dermo-Cre mice, indicated a delay in the fracture healing, accompanied by less formation of fracture callus. Fracture healing is considered to be a regenerative process consisting of many phases, each of which involves the formation of a variety of tissue types [22]. The formation of fracture callus appears during the secondary phase bone healing and imparts mechanical stability to the healing fracture [23]. A previous study has uncovered that mice constitutively lacking epithelial Cbfb exhibited short incisors, marked underdevelopment of the cervical loop, and diminished expression of epithelial Fgf9 expression and mesenchymal Fgf3 and Fgf10 in the cervical loop [24]. Therefore, Cbfb might actively participate in the processes of skeletal development as well as fracture healing.

The Cbfb knockout mice were observed to exhibit downregulated PCNA and collagen II expression. Collagen II, which is known to regulate chondrogenesis of mesenchymal stem cells, demonstrably has the potential to facilitate osteogenesis and suppress adipogenesis during early stages of mesenchymal stem cell differentiation [25]. Stegemann et al. also revealed collagen II to be a key factor in enhancing chondrogenic differentiation in agarose-based modular microtissues [26]. PCNA is widely accepted to be involved in distinct pathways of DNA post-replication repair [20, 27]. In addition to DNA repair, PCNA also plays a key role in other fundamental cellular processes, such as chromatin remodeling, sister chromatid cohesion, and cell cycle control [28]. In the presence of Cbfb knockdown, the differentiation of chondrocytes and osteoblasts is observed to be severely inhibited in vitro [15]. Hence, knockout of Cbfb can result in conspicuously increased expression of PCNA and collagen II.

Additionally, our findings indicated that Cbfb knockout can inhibit ALP activity and mineralization of osteoblasts. Extracellular matrix mineralization represents an essential physiological process in the formation of teeth and bones, as well as in growth plate cartilage formation during skeletal growth [29]. ALP activity is known to be a serum biochemical marker of bone formation and has been verified to be a clinically useful tool in predicting fractures with a risk of nonunion and for monitoring the progress of healing [30]. Diminished ALP activity and mineralization in the MC3T3-E1 osteoblast-like cell line can result in delayed osteoblast differentiation [31]. IL-6 is an important factor in the early stages of fracture healing, and mice with IL-6 knockout presented with delayed callus maturation, mineralization, and remodeling during fracture healing when compared to the callus of WT mice [32].

Cbfb knockout disrupted osteoblast differentiation, which was evidenced by decreased expression of ALP, Bglapl, Runx2, and SPPL as well as the inhibited protein expression of Osteocalcin, Atf4, and Osterixin in Cbfb knockout mice. On the other hand, BrdU results showed no effects of Cbfb knockout caused on osteoblast proliferation. However, the expression of the chondrocyte maturation markers Runx2, Osterix, and Osteopontin was significantly reduced in Cbfb knockout mice relative to the WT mice [33]. Another study revealed that microRNA-145 physiologically regulates bone formation and osteoblast differentiation by forming a regulatory microRNA network through altered Cbfb expression [11]. Moreover, in addition to facilitating chondrocyte differentiation for the growth and maintenance of the skeleton in postnatal mice, Cbfb also promotes osteoblast differentiation [10].

## Conclusion

The key findings obtained from the present study confirm our prediction that Cbfb could potentially stimulate osteoblast differentiation and fibula fracture healing. Furthermore, Cbfb emerges a new biomarker and target for treatment of fibula fracture. As we are uncovering the Cbfb-mediated mechanisms in osteoblast differentiation, new strategies and targets may emerge for therapies that prevent and treat fractures. Under the experiment conditions, Cbfb conditional knockout may be affected by various factors, such that the generalization of experimental results calls for some caution. Further studies are required to understand fully the specific molecular mechanistic actions of Cbfb conditional knockout, in advance of clinical translation of this therapeutic approach.

## Supplementary Information

**Additional file 1: Supplementary figure 1.** Schematic view of the modeling process and the X-rays at each time point. A, the modeling process of WT mice at days 1, 14, and 28 following fracture; B, the modeling process of Cbfbf/f; Dermo-Cre mice at days 1, 14, and 28 following fracture.

**Additional file 2: Supplementary Table 1.** Primer sequences for reverse transcription quantitative polymerase chain reaction.

## Data Availability

The datasets generated during and/or analyzed during the current study are available from the corresponding author on reasonable request.

## Abbreviations

Cbfb: Core-binding factor beta
PCNA: Proliferating cell nuclear antigen
ALP: Alkaline phosphatase
WT: Wild type
SP: Streptavidin-perosidase
PCNA: Proliferating cell nuclear antigen
EDTA: Ethylenediamine tetraacetic acid
PVDF: Polyvinylidene fluoride
ECL: Enhanced chemiluminescence
