# Supplementary figures and images for: Core-binding factor beta is required for osteoblast differentiation during fibula fracture healing
Source: J Orthop Surg Res. 2021 May 14;16:313. doi: 10.1186/s13018-021-02410-9 (PMC8120848; doi:10.1186/s13018-021-02410-9)

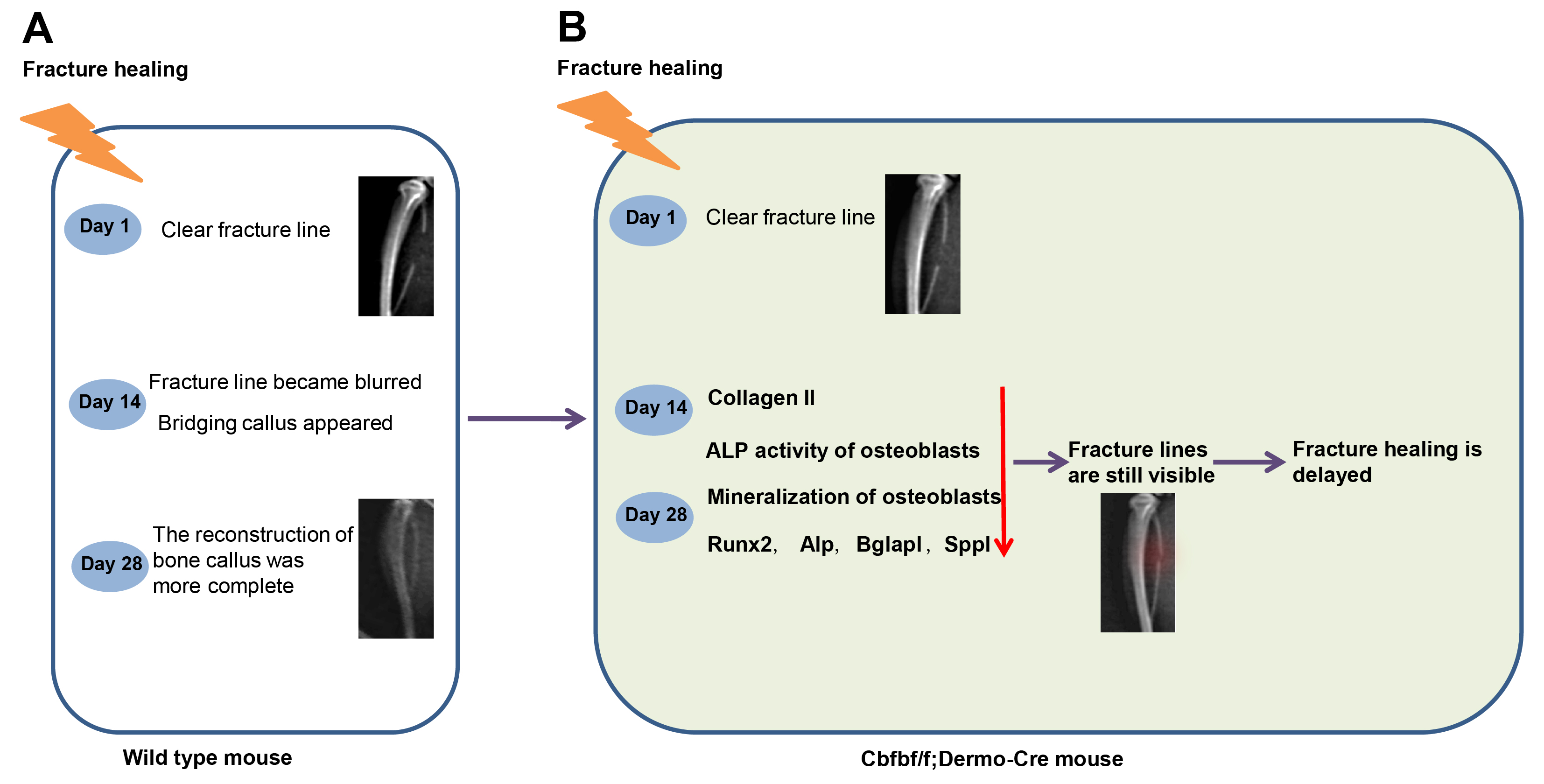

Supplement: Supplementary file 1 — Additional file 1: Supplementary figure 1. Schematic view of the modeling process and the X-rays at each time point. A, the modeling process of WT mice at days 1, 14, and 28 following fracture; B, the modeling process of Cbfbf/f; Dermo-Cre mice at days 1, 14, and 28 following fracture. [file 13018_2021_2410_MOESM1_ESM.jpg]
