# Supplementary material for: Core-binding factor beta is required for osteoblast differentiation during fibula fracture healing
Source: J Orthop Surg Res. 2021 May 14;16:313. doi: 10.1186/s13018-021-02410-9 (PMC8120848; doi:10.1186/s13018-021-02410-9)
Supplement: Supplementary file 2 — Additional file 2: Supplementary Table 1. Primer sequences for reverse transcription quantitative polymerase chain reaction. [file 13018_2021_2410_MOESM2_ESM.docx]

**Supplementary Table 1** Primer sequences for reverse transcription quantitative polymerase chain reaction

| Gene | Forward sequence | Reverse sequence | Products |
| --- | --- | --- | --- |
| Bglapl | CTGACCTCACAGATCCCAAGC | TGGTCTGATAGCTCGTCACAAG | 187bp |
| ALP | GTGACTACCACTCGGGTGAAC | CTCTGGTGGCATCTCGTTATC | 96bp |
| Cbfb | GATCATGAGCCCTTTTTCTCC | GGCAAAAGCAATCTGGTAGC | 175bp |
| Runx2 | GACTGTGGTTACCGTCATGGC | ACTTGGTTTTTCATAACAGCGGA | 84bp |
| Osteocalcin | GAAGCCCAGCGGTGCA | CACTACCTCGCTGCCCTCC | 103bp |
| ATF4 | TGGCTGGCTGTGGATGG | TCCCGGAGAAGGCATCCT | 72bp |
| Osterix | CCCCACCTCTTGCAACCA | CCTTCTAGCTGCCCACTATTTCC | 89bp |
| GAPDH | TTCACCACCATGGAGAAGGC | GGCATGGACTGTGGTCATGA | 100bp |
